# Temporal changes in brain morphology related to inflammation and schizophrenia: an omnigenic Mendelian randomization study

**DOI:** 10.1017/S003329172400014X

**Published:** 2024-07

**Authors:** Yunjia Liu, Hongyan Ren, Yamin Zhang, Wei Deng, Xiaohong Ma, Liansheng Zhao, Xiaojing Li, Pak Sham, Qiang Wang, Tao Li

**Affiliations:** 1 Mental Health Center and Psychiatric Laboratory, The State Key Laboratory of Biotherapy, West China Hospital of Sichuan University, Chengdu, Sichuan, China; 2 Nanhu Brain-computer Interface Institute, Hangzhou 311100, China; 3 Affiliated Mental Health Center & Hangzhou Seventh People's Hospital and School of Brain Science and Brain Medicine, Zhejiang University School of Medicine, Hangzhou, 310058, China; 4 Lingang Laboratory, Shanghai 200031, China; 5 Liangzhu Laboratory, MOE Frontier Science Center for Brain Science and Brain-machine Integration, State Key Laboratory of Brain-machine Intelligence, Zhejiang University, 1369 West Wenyi Road, Hangzhou 311121, China; 6 State Key Laboratory of Brain and Cognitive Sciences, Centre for Genomic Sciences, and Department of Psychiatry, Li Ka Shing Faculty of Medicine, The University of Hong Kong, Pokfulam, Hong Kong SAR, China; 7 NHC and CAMS Key Laboratory of Medical Neurobiology, Zhejiang University, Hangzhou 310058, China

**Keywords:** brain morphology, inflamattion, Mendelian randomization, schizophrenia

## Abstract

**Background:**

Over the past several decades, more research focuses have been made on the inflammation/immune hypothesis of schizophrenia. Building upon synaptic plasticity hypothesis, inflammation may contribute the underlying pathophysiology of schizophrenia. Yet, pinpointing the specific inflammatory agents responsible for schizophrenia remains a complex challenge, mainly due to medication and metabolic status. Multiple lines of evidence point to a wide-spread genetic association across genome underlying the phenotypic variations of schizophrenia.

**Method:**

We collected the latest genome-wide association analysis (GWAS) summary data of schizophrenia, cytokines, and longitudinal change of brain. We utilized the omnigenic model which takes into account all genomic SNPs included in the GWAS of trait, instead of traditional Mendelian randomization (MR) methods. We conducted two round MR to investigate the inflammatory triggers of schizophrenia and the resulting longitudinal changes in the brain.

**Results:**

We identified seven inflammation markers linked to schizophrenia onset, which all passed the Bonferroni correction for multiple comparisons (bNGF, GROA(CXCL1), IL-8, M-CSF, MCP-3 (CCL7), TNF-*β*, CRP). Moreover, CRP were found to significantly influence the linear rate of brain morphology changes, predominantly in the white matter of the cerebrum and cerebellum.

**Conclusion:**

With an omnigenic approach, our study sheds light on the immune pathology of schizophrenia. Although these findings need confirmation from future studies employing different methodologies, our work provides substantial evidence that pervasive, low-level neuroinflammation may play a pivotal role in schizophrenia, potentially leading to notable longitudinal changes in brain morphology.

## Introduction

Schizophrenia (SCZ) is a complex disorder characterized by a genetic architecture that includes polygenicity and pleiotropy (Ripke et al., 2013; Schizophrenia Working Group of the Psychiatric Genomics, 2014). Recent advances in genotyping and sequencing technology, alongside multinational collaborations for sample collections as epitomized by Psychiatric Genomic Consortium (PGC), have facilitated genome-wide scans for risk variants of SCZ in a well-powered sample. These developments have provided fresh insights into the architecture and pathogenesis of SCZ. However, mapping the complete genetic architecture underlying the complex disease like SCZ and translating the existing GWAS results remains elusive, the methods such as polygenic risk score only involves variants passing certain significance thresholds and can only explain a small proportion of phenotypic variation, a problem dubbed ‘the missing heritability’ (Zuk, Hechter, Sunyaev, & Lander, 2012). In a groundbreaking study, Boyle et al., proposed an omnigenic model, suggesting that in disease-relevant cell types, essentially all genes contribute to the condition (Boyle, Li, & Pritchard, 2017). Concurrently, three waves of genome-wide association studies (GWASs) by the PGC indicated a potentially critical role for inflammation/infection in the pathophysiological mechanism of SCZ, with the most associated genomic regions highlighting the MHC region on chromosome 6 (Pardiñas et al., 2018; Schizophrenia Working Group of the Psychiatric Genomics, 2014; Trubetskoy et al., 2022).

Despite abundant evidence supporting inflammation's role in SCZ (Khandaker et al., 2015; Sellgren et al., 2021), determining specific inflammatory markers indicative of SCZ remains challenging. The most detectable and widespread inflammatory molecules in the peripheral blood system are cytokines and chemokines, which maintain immune cell homeostasis and coordinate immune responses (Ramesh, MacLean, & Philipp, 2013). Recent conceptual shifts have reframed the relationship between the brain and the immune system. It is now accepted that the brain is not immune-privileged and that brain cells like neurons and microglia release cytokines essential for both normal function and disease states (Kwon, 2022). In the context of schizophrenia, researchers have found evidence of cytokines such as C-reactive protein (CRP), IL-1*β*, IL-6, IL-12, interferon-*γ*, and tumor necrosis factor *α* (TNF-*α*) being associated with endophenotypes and varying disease status of schizophrenia (Lesh et al., 2018; Lin et al., 2021).

While promising, these findings are not always consistent due to methodological heterogeneity and moderate study sample sizes. Other factors can also confound the investigation of cytokine levels in the peripheral blood in schizophrenia, including antipsychotics, metabolites, comorbid inflammatory conditions, and age (Decker, Gotta, Wellmann, & Ritz, 2017; Himmerich, Patsalos, Lichtblau, Ibrahim, & Dalton, 2019). Furthermore, ongoing debates exist regarding the causal inference of regression analysis, the most commonly used statistical method in association studies. Linear regression implies causality only if the covariates are from a controlled experiment and the experiment isolates the hypothesized causal factor well (Constantine, 2012). Unfortunately, most association studies with cytokine levels as covariates were conducted in a non-experimental controlled manner. Mendelian randomization (MR) provides a useful tool for detecting causal relationships between phenotype pairs, generating a more robust causal estimate due to the immutability of genotypes as instrument variables (IVs, randomization at birth) (Sanderson et al., 2022). Two-sample MR, which utilizes publicly accessible summary-level data from GWAS, has gained popularity in recent years due to free access to a vast resource of GWAS results of various phenotypes or traits (Hemani et al., 2018). Unlike one-sample MR, two-sample MR does not require the effect of the IV-exposure association and the IV-outcome factor association to be collected from the same participant sample, potentially avoiding confounding factors such as the ‘winner's curse’ (Zou et al., 2022).

**Figure 1.**
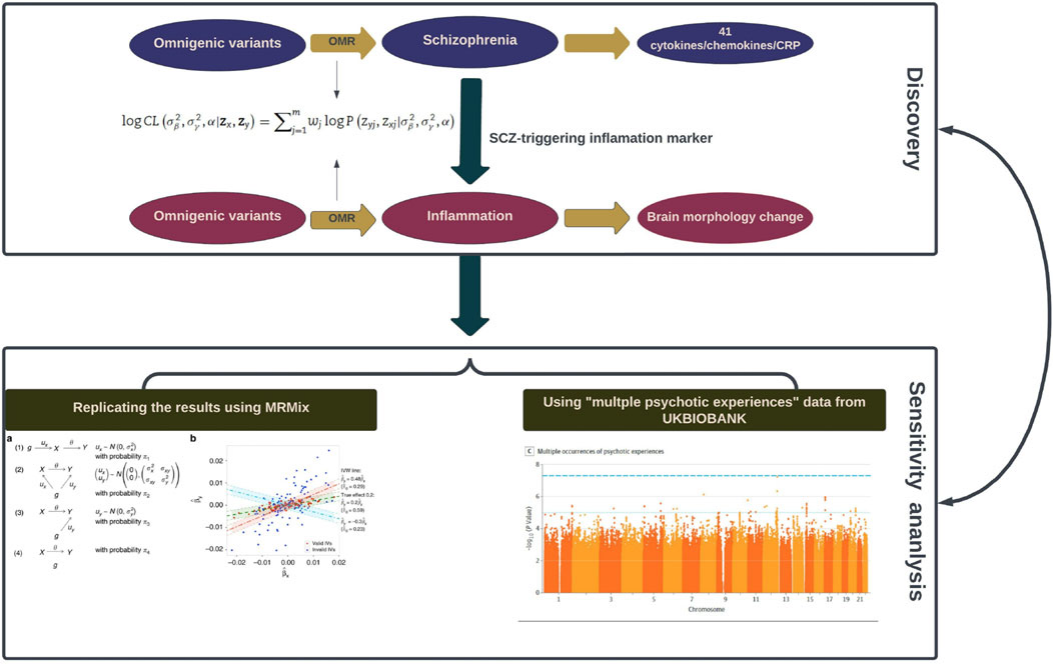
Schematic illustration of the analysis framework in our study. Two black rectangles demonstrate the two sequential phases: the initial ‘Discovery Phase’ (the upper one) involves the utilization of omnigenetic schizophrenia (SCZ) genotypes (IVs) to identify the inflammation markers with a potential causal relationship to SCZ. Moving to the second ‘Replication Phase’ (the lower one), the same omnigenetic IVs associated with SCZ-triggering inflammation markers were employed to identify the brain regions exhibiting causal linear morphological changes. Concurrently, for each MR analysis, we calculated the degree of horizontal pleiotropy effect on outcome measures. The lower panel portrays the methods employed to replicate our main results. The left side gives an overview of the MRMix algorithm, while the right exhibits a Manhattan plot detailing multiple psychotic experiences used in the replication phase.

A previous two-sample MR study by Hartwig et al., suggests a negative (protective) effect of CRP and a risk-increasing (positive) effect of sIL-6R (potentially mediated at least in part by CRP) on the lifetime risk of schizophrenia. (Hartwig, Borges, Horta, Bowden, & Davey Smith, 2017). However, their study had several limitations. Following the conventional hypothesis of MR, the study by Hartwig et al., chose the SNPs, the association of which reached the genome-wide significance, to be IVs in the causal inference of MR. However, many previous studies showed that even the SNPs with large effect-sizes could only explain a small proportion of phenotypic variation, thus under-estimating the MR estimate (Davies, Holmes, & Davey Smith, 2018). The current study, taking into account the genetic architecture of complex diseases such as SCZ and assuming an omnigenic model in which essentially all genes contribute to the phenotypic variation of complex traits, includes all genome-wide SNPs as IVs to answer two questions: (1) What does the pathogenesis of schizophrenia trigger the immune response in terms of inflammatory molecules, including cytokines, chemokines and CRP (SCZ-triggering inflammatory markers)? The ENIGMA (Enhancing NeuroImaging Genetics through Meta-Analysis) consortium recently highlighted genetic variants associated with longitudinal changes in brain structure (Brouwer et al., 2022), and various studies have identified systemic inflammation as a predictor of brain aging (Corlier et al., 2018), which leads to our second question: (2) What is the impact of SCZ-triggering inflammatory markers on longitudinal brain changes? (Fig. 1).

## Materials and methods

### Data source

The MR analysis of SCZ and inflammation in our study utilized, as the exposure, the summary statistics of the schizophrenia GWAS conducted by Trubetskoy et al., which comprised 76 755 cases and 243 649 controls of European ancestry (Trubetskoy et al., 2022). For the inflammation outcomes, we used the summary statistics of GWAS of 41 cytokines/chemokines in the peripheral blood of 8293 healthy volunteers of European ancestry (Kalaoja et al., 2021) (online Supplementary Table S2). For CRP, we selected the summary statistics of the latest CRP GWAS conducted on 427 367 participants of European descent (Said et al., 2022). No significant sample overlapping was observed within the three datasets (SCZ, cytokines/chemokines, and CRP).

The SCZ-triggering inflammatory markers identified in the first analysis were taken to a second MR analysis of inflammation and longitudinal morphology change in fifteen brain phenotypes, including the amygdala, caudate, cerebellum gray matter, cerebellum white matter, cerebral white matter, cortical gray matter, hippocampus, lateral ventricles, cortical mean thickness, nucleus accumbens, pallidum, putamen, surface area, thalamus, and total brain volume. For the outcome measure, we chose the GWAS summary statistics of longitudinal changes (following-up period no less than six months) in brain structure conducted by the ENIGMA consortium in a sample of 15 640 individuals of European descent (Brouwer et al., 2022). There was no known sample overlapping between these two datasets. The details of all the summary data included in the analysis were listed in online Supplementary Table S1.

### Two-sample MR analysis

Given easy access to GWAS results, numerous methods have been developed for two-sample MR analysis. Yet, most of these methods require (1) using SNPs with significant association at the genome-wide level as IVs and (2) the selected IVs are independent of each other, i.e. in linkage equilibrium (LD). However, schizophrenia, like many other complex diseases/traits, is a polygenic and even omnigenic disease. Furthermore, the independent IVs in a less perfect LD with the true causal ones could lead to power loss. To tackle these issues and increase the power of causal inference, Lu et al., based on the concept of the omnigenic model, developed a new analysis framework for two-sample MR studies called omnigenic Mendelian randomization (OMR) (Wang, Gao, Fan, Xue, & Zhou, 2021). The OMR relaxes the relevance and independence assumptions by including all genome-wide SNPs as IVs and explicitly controlling for the potential horizontal pleiotropy by including an additional term, G_y_*γ*, in the equation below.1
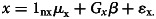
2
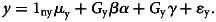
In the equation above, 1 represents a vector of 1's with the corresponding dimensionality shown in the subscript, *μ*_x_ is the intercept for the exposure model; *μ*_y_ is the intercept for the outcome model; nx and ny are the sample size of GWASs for exposure measure and outcome measure, respectively; *β* is the genotype effect on the exposure variable; *γ* is the horizontal pleiotropy effect of SNPs on the outcome variable; *α* is a scalar that represents the causal effect of the exposure variable on the outcome variable; *x* and *y* are residual error vectors of size nx and ny, respectively. The OMR constructs a log composite likelihood as a weighted combination of the log marginal likelihood. Both simulation and real dataset analysis showed that, compared with other methods, OMR showed superiority in controlling type I error and horizontal pleiotropy effect across different genetic architectures.

We used the R package ‘OMR’ to perform the omnigenic MR analyses. To harmonize the data, we implemented the quality control and merged two data sets with reference panel from the 1000 Genomes Project Phase3. Each OMR analysis output includes two primary parameters: the causal effect (*α*) and the proportion of SNP horizontal pleiotropy effect in the outcome variable (*γ*). Figure 1 displays the analysis framework of this study. We defined SCZ-triggering inflammatory markers as ones with a significant *p* value surviving the Bonferroni multiple comparison correction (0.05/42 = 0.00119). These markers were then chosen as the exposure in the second round of MR analysis of inflammation ⇒ longitudinal morphology change of the brain, with the *p* value being corrected for Bonferroni multiple comparisons (0.05/(15 × 7) = 0.000477).

### Replication analysis

To evaluate the robustness of our main results, we carried out a two-layered replication analysis. For the first layer, we chose a different MR method, ‘MRMix’, a multiple-variant approach (Qi & Chatterjee, 2019), to re-run the analysis on the inflammation markers showing a significant causal link with longitudinal brain changes in the previous step. For the second layer, we selected a different dataset, GWAS of multiple psychotic experiences, to replicate the primary results of SCZ ⇒ inflammation (Legge et al., 2019).

## Results

### Seven inflammation markers showing a significant change in response to schizophrenia

**Figure 2.**
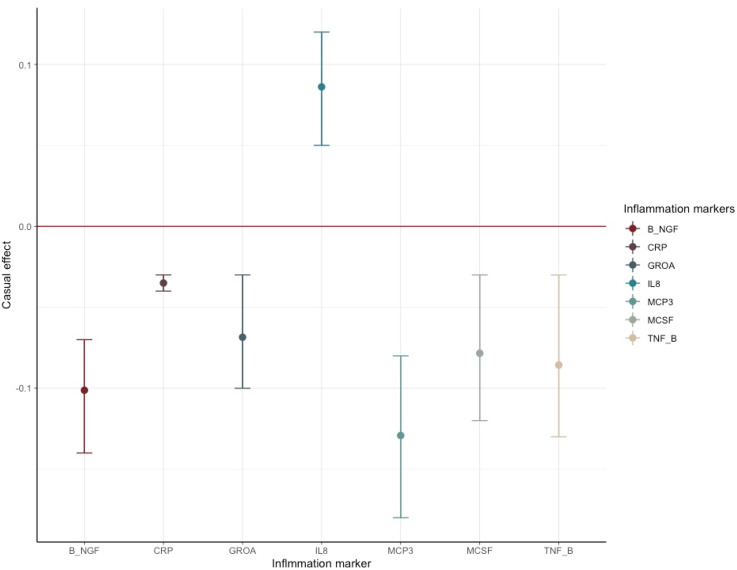
Causal estimates and corresponding 95% confidence intervals of seven inflammation markers that generated significant *p* values in the MR analysis of schizophrenia ⇒ inflammation. The red line denotes no causal effect between schizophrenia and inflammation markers. The vertical bars above the red line represent inflammation markers whose level increases by one standard unit for each additional standard deviation (s.d.) in the risk of schizophrenia. Conversely, the bars below the red line signify estimates of inflammation markers whose level decrease for each s.d. increase in the risk of schizophrenia.

The results of SCZ ⇒ inflammation analyses are summarized in Table 1 and Fig. 2. Omnigenic IVs of SCZ led to the change of seven inflammation markers in the peripheral blood. The most significant was CRP, with a causal estimate of −0.035 [−0.04 to −0.03] (*α*, causal effects with 95% CI) in response to schizophrenia (*p value* = 1.71 × 10^−32^), and approximately 0.1% of SNPs involved indicated horizontal pleiotropy. The second most significant inflammation marker, bNGF (basic nerve growth factor), decreased by 0.10 in the peripheral blood as the quantitative risk of SCZ increased with each standard deviation (s.d.) unit (bNGF: *α* = −0.101 [−0.136 to −0.066], *p value* = 1.45 × 10^−8^). We observed systematic immunological deficits in the inflammatory response to SCZ, with only IL-8 exhibiting an elevated level in response to schizophrenia (IL-8: *α* = 0.086 [0.053–0.119], *p value* = 3.64 × 10^−7^). Interestingly, GROA (CXCL1), which could bind to the same receptor as IL-8 and CXCR2, was also significantly involved, with its mean value decreasing by 0.068 as the risk for SCZ increased each s.d. unit, implicating the role of the IL-8 pathway in the inflammatory mechanism of SCZ (GROA: *α* = −0.068 [−0.103 to −0.034], *p value* = 1.15 × 10^−4^).

**Table 1.** Causal estimates of six cytokines/chemokines and CRP in MR analysis of SCZ ⇒ inflammation using OMR

Inflammation markers	*α*		*β*	*γ*	*Z*	*p*
	Causal effects	95% CI				
bNGF	−0.101	(−0.14 to −0.07)	0.005	0.001	−5.667	1.45 × 10^−8^
GROA (CXCL1)	−0.068	(−0.10 to −0.03)	0.005	0	−3.857	1.15 × 10^−4^
IL-8	0.086	(0.05 to 0.12)	0.005	0.002	5.087	3.64 × 10^−7^
M-CSF	−0.078	(−0.12 to −0.03)	0.005	0	−3.311	9.30 × 10^−4^
MCP-3 (CCL7)	−0.129	(−0.18 to −0.08)	0.005	0.004	−4.840	1.3 × 10^−6^
TNF-*β*	−0.086	(−0.13 to −0.04)	0.005	0	−3.566	3.63 × 10^−4^
CRP	−0.035	(−0.04 to −0.03)	0.005	0.002	−11.869	1.71 × 10^−32^

For horizontal pleiotropy, most causal estimates of identified markers, except those of GROA (CXCL1) and M-CSF, indicated varying yet relatively modest degrees of horizontal pleiotropy, signaling a potentially exclusive pathway linking SCZ to these markers.

### CRP as the SCZ-triggering inflammation marker conferring impact on the longitudinal morphology change of different brain regions

**Figure 3.**
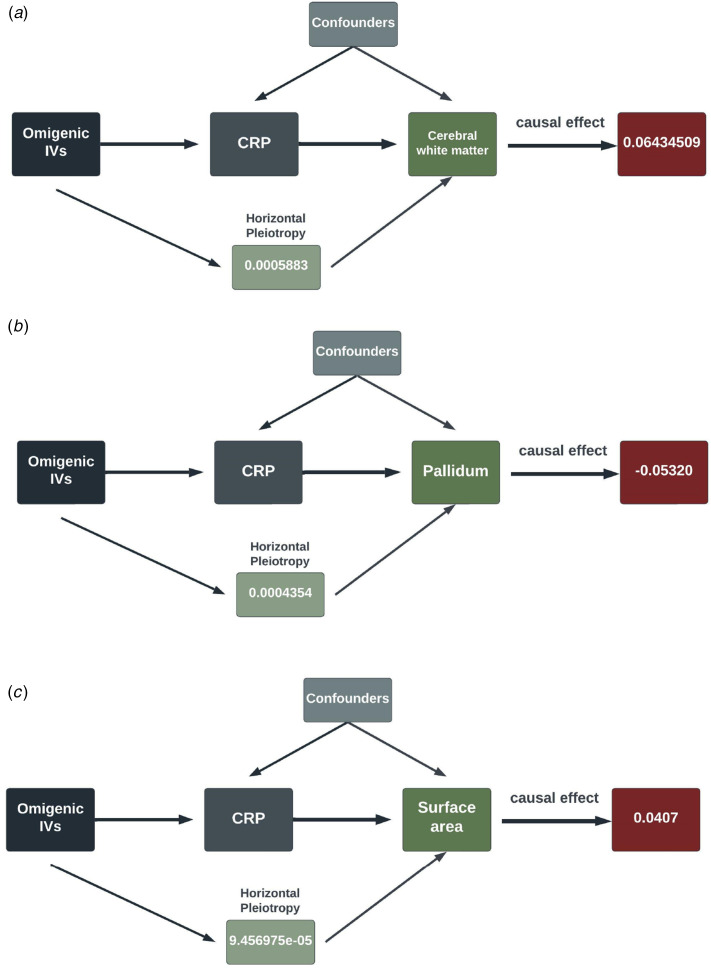
The schematic diagrams illustrating the causal effect of significant SCZ-triggering inflammation markers, CRP, on linear morphology change within different brain regions. Each diagram contains the causal effect estimates. In addition to these causal estimates, we also calculated the impact of horizontal pleiotropy, generated by the omnigenic IVs, on the morphological changes within the brain. (IVs, instrumental variables; CRP, C-reactive Protein; WM, white matter).

In the second round of MR analysis of inflammation ⇒ the longitudinal brain changes, three out of seven SCZ-triggering inflammation markers, GROA (CXCL1), IL-8 and CRP, caused the significant longitudinal change in the different brain regions. Specifically, CRP led to the most significant and wide-ranging linear change in the brain morphology: one standard unit increase of CRP in the peripheral blood could lead to an increase in the mean change rate of the cerebral white matter by 0.064 [0.396–0.089] (*p value* = 3.588 × 10^−7^), a reduction in the change rate of pallidum by 0.053 [0.034–0.072] (*p value* = 4.02 × 10^−8^), and an increase in change rate by 0.041 [0.020–0.061] in the surface area (*p value* = 8.67 × 10^−5^). CRP also caused a decrease in the mean linear change rate by 0.033 [0.014–0.052] in the white matter of cerebellum, with a *p value* falling short of multiple comparison corrections (*p value* = 6.7 × 10^−4^). Additionally, at a nominally significant level, we also detected some probably causal relationship. A decrease in linear change rate of lateral ventricles (LCs) mean value was found associated with an increased rate of 0.204 [0.042–0.366] of GROA (*p value* = 0.01). IL-8 was casually associated with the linear change rate of cerebral white matter (*α* = −0.253 [−0.43 to −0.07], *p value* = 0.0064) and amygdala (*α* = −0.528 [−0.961 to −0.096], *p value* = 0.017)). The findings are summarized in the pathway diagram in Fig. 3.

**Figure 4.**
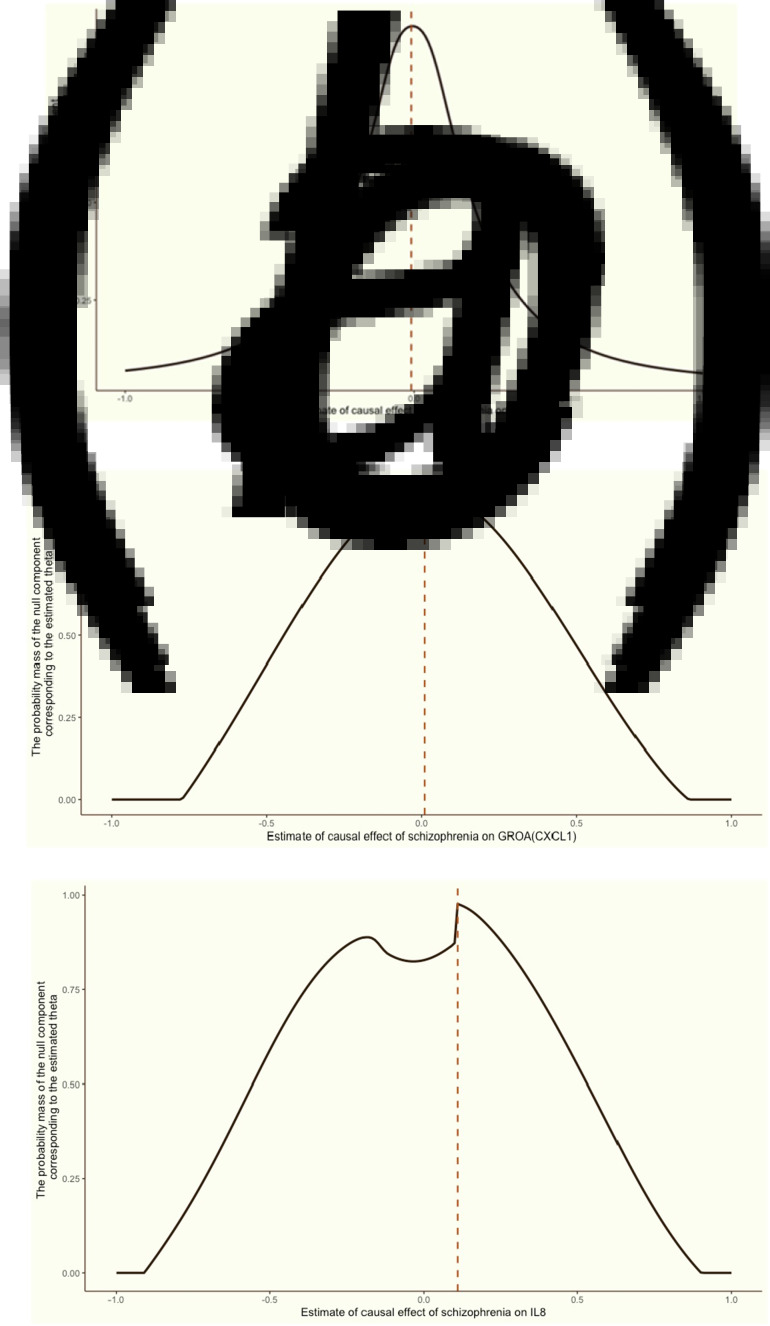
Grid search results for the causal effects (*θ*) of schizophrenia on CRP, GROA (CXCL1) and IL-8. We aimed to discern the causal effect (*θ*) of schizophrenia on CRP (a), GROA (b), and IL-8 (c). The vertical red dashed line in each graph indicates the causal estimates corresponding to the probability mass's peak.

Replication using MRMix validated the significant causal relationship of schizophrenia to the three inflammation markers mentioned above. As displayed in Fig. 4, the direction of the causal effect arising from MRMix was consistent with those from OMR for all markers except for GROA (CXCL1), which generated a positive estimate of 0.01 instead of a negative causal effect. Using the GWAS summary statistics of multiple psychotic experiences from the UK biobank, we identified the causal relationship between multiple psychotic experiences and CRP as nominally significant (*α* = −0.01, *p value* = 0.045).

## Discussion

In an innovative approach, our study employed an omnigenic model to elucidate the immunological aspects of schizophrenia and their effect on longitudinal changes in brain morphology. We found several inflammatory markers associated to schizophrenia, indicating a systematic immunological imbalance in schizophrenia. Notably, we discovered that three SCZ-triggering markers, CRP, GROA(CXCL), and IL-8, significantly influence the linear change rate of brain structures, especially the white matter. The subsequent analysis using MRMix confirmed our primary findings for all three markers, and we also discerned the causal implication of CRP through the ‘multiple psychotic experiences’ results.

The key findings of our study include the causal relationship between schizophrenia and CRP and a directed relationship between CRP and the linear change rate in multiple brain regions. The former finding is partially consistent with the study by Hartwig et al. (Hartwig et al., 2017). While Hartwig et al., suggests a negative (protective) effect of CRP on the lifetime risk of schizophrenia, our study implies that as the quantitative risk of SCZ increased by one standard unit, the mean value of CRP decreased by 0.035. Notably, as the quantitative risk of SCZ increased by one standard unit, the mean value of CRP decreased by 0.035. CRP, an acute-phase protein predominantly secreted by hepatocytes, can also be produced by neurons in response to infection (Yasojima, Schwab, McGeer, & McGeer, 2000). Presenting either as a pentameric form (pCRP) or as individual monomers at sites of inflammation (CRP), our findings echo prior research, suggesting a low-grade yet continuous anomaly in CRP is fundamental to the pathogenesis of SCZ, such as intrauterine influences and stress, lead to immunological deficits. These deficits are exemplified by the reduction in CRP level observed in our study.

These immunological deficits, combined with later-life environmental factors, could precipitate the onset of schizophrenic symptoms. Moreover, our subsequent MR analysis indicated that CRP significantly contributes to deviant longitudinal morphological changes in various brain regions, especially white matter volume (WMV). Prior research has proposed that inflammation could cause abnormalities in white matter, including lesions (Wu, 2012) and WMV changes (Raz, Yang, Dahle, & Land, 2012). Our findings estimated an increase in cerebral white matter mean value and a decrease in cerebellar WMV mean value as CRP increases. As per a foundational study by Bethlehem et al., WMV increased rapidly from mid-gestation to early childhood, peaking in young adulthood, which coincides with the typical onset age of schizophrenia (Bethlehem et al., 2022). Our results suggest that longitudinal changes in CRP levels caused by SCZ lead to divergent growth trajectories of WMV in cerebral and cerebellar areas. Our study also established the causal link from CRP to the pallidum's GMV longitudinal changes, which play a significant role in various mental functions (Klaassen, Heiniger, Vaca Sánchez, Harvey, & Rainer, 2021; Takeuchi et al., 2017). Considering the omnigenic model, maybe we could cautiously speculate that the local region is influenced by the core genes and the whole cerebral white matter by the peripheral genes.

Interestingly, Weiqiu et al., identified a notable shared genetic architecture between schizophrenia and subcortical volumes, precisely 54% for schizophrenia. Consistent with our hypothesis, they also found that shared genetic loci peaked at the prenatal age, suggesting that the pathogenesis of schizophrenia, especially immunological aspects, could start around the perinatal period, potentially triggered or exacerbated by intrauterine infection or inflammation involving CRP (Cheng et al., 2022). Moreover, we could further infer that synaptic plasticity play a vital role in the underlying pathophysiology. As in the previous PGC3 SCZ GWAS, risk genes are expressed in the both excitatory and inhibitory of neurons, which is also associated with inflammation (de Bartolomeis et al., 2022).

Our study also identified another SCZ-triggering inflammatory marker, GROA (CXCL1), which could lead to a decreased linear change rate in the lateral ventricle. GROA, structurally similar to interleukin-8 (IL-8), has potent neutrophil-stimulating activity (Geiser, Dewald, Ehrengruber, Clark-Lewis, & Baggiolini, 1993). Several studies have demonstrated its significant differences in both gene expression and DNA methylation between patients with SCZ and healthy controls, as well as among different subtypes of schizophrenia (Zhou et al., 2018). In the CNS, CXCL1 primarily binds to CXCR2 and promotes the proliferation of neuronal stem cells (NSCs) in both vitro and vivo, especially in the subventricular zone of the lateral ventricle (Shang et al., 2019). Our analysis generated a negative causal estimate, hinting at the possible suppression effect of SCZ on the GROA (CXCL1) level and subsequently on the proliferation and migration of NSCs.

We also found IL-8, another SCZ-triggering inflammation marker, to influence the linear change rate of brain morphology. IL-8, a crucial mediator of the immune response, is typically synthesized by glial cells and astrocytes in the CNS (Atta-ur-Rahman, Harvey, & Siddiqui, 1999; Ehrlich et al., 1998). Our analysis of SCZ and inflammation indicated a positive causal effect of schizophrenia on IL-8, implying that schizophrenia could lead to increased IL-8 level, a finding in line with previous studies (Boerrigter et al., 2017; Brown et al., 2004). Furthermore, we discovered that IL-8 results in a decreased change rate of cerebral white matter. Potential roles IL-8 could play in neuroinflammation include facilitating the synthesis of pro-apoptotic protein, inducing the gene expression of pro-inflammatory proteases with neurotoxic properties, and recruitment of immune cells to the inflammation sites (Ramesh et al., 2013). Of note, both IL-8 and GROA (CXCL1) belong to the same CXC group, which could bind to CXCR2 with high affinity (Waugh & Wilson, 2008). Giovannelli et al., using the patch clamp technique and laser confocal microscopy, demonstrated that IL-8 and GROA could modulate the activity of Purkinje neurons in the mouse cerebellum (Giovannelli et al., 1998). Besides, our study indicated that IL-8 and GROA could cause significant longitudinal changes in cerebral white matter and lateral ventricle. A neuroimaging study found a strong association between white matter diffusivity and ventricle volume in patients with Alzheimer's disease (AD) (Coutu, Goldblatt, Rosas, Salat, & Alzheimer's Disease Neuroimaging, 2016), which strongly suggests that further investigations are warranted to unravel the cellular mechanism of the IL-8 pathway-mediated effect on cerebral white matter in the schizophrenia.

Additionally, our study identified that while the peripheral level of bNGF, M-CSF, MCP-3 and TNF-*β* could be altered by schizophrenia, they were not found to be causally associated with the linear change rate of brain morphology. bNGF is a neurotrophic factor that regulates the functions of differentiated neurons (Aloe, Rocco, Balzamino, & Micera, 2015). Prior studies found its dysfunction in schizophrenia, especially its role in the structural deviation of brain, including areas highly involved in crucial cognitive functions such as the prefrontal and temporal cortex. Our findings of decreased bNGF level suggest a deficit neurodevelopmental process preceded or precipitated by inflammation or infection. M-CSF, macrophage colony-stimulating factor, is a widely expressed cytokine that stimulates progenitor cells from bone marrow and subsequent development, proliferation and maintenance of mononuclear phagocytes such as dendritic cells and microglia (Hume & MacDonald, 2012). M-CSF, along with anti-inflammatory factors such as TNF-*β*, can polarize microglia towards anti-inflammatory (M2) directions (Pons & Rivest, 2018). Both M-CSF and TNF-*β* showed lower levels in response to the pathogenesis of schizophrenia, hinting at a potential early-onset dysfunction in restricting neuroinflammation.

MCP-3 (CCL7), the monocyte chemotactic protein 3, belongs to the MCP subgroup of CXC chemokines and is a potent chemoattractant for various leukocytes, including dendritic cells (DCs) (Liu, Cai, Liu, Wu, & Xiong, 2018). Cathomas et al., found a significantly lowered level of CCL7 in patients with SCZ, consistent with our estimates (Cathomas et al., 2021). We did not find any causal relationship between these five cytokines and the linear change in brain morphology, and it is theoretically possible that they might exert their effects through other pathophysiological processes, possibly in a time-specific manner.

While our study, to the best of our knowledge, is the first study to utilize an omnigenic conceptual framework to elucidate the inflammatory implications of schizophrenia and the influence of these inflammatory factors on the temporal change in brain morphology, we are fully aware of its limitations. Despite efforts to validate the robustness of our findings by replicating initial results with a different methodology and dataset, several constraints remain. First, we could not eliminate all potential confounders in our MR analyses, thereby possibly violating the exclusion restriction assumption. Given the inherent polygenic or omnigenic nature of complex diseases and traits, the human genome has widespread pleiotropy. Consequently, it is currently impractical to identify and account for all potential confounding factors. It should also be noted that most variants are common but with less disease specificity in the GWAS summary data. Secondly, our study only carried out a unidirectional MR analysis. We refrained from exploring the causality of a cytokine to schizophrenia out of concern that the instrumental variables (IVs) for cytokines would be severely biased towards under-powering due to the modest sample size of cytokine GWAS.

## Conclusion

In conclusion, leveraging an omnigenic conceptual framework, we explored the inflammatory mediators of schizophrenia and their impact on the temporal longitudinal change in brain morphology. Our results spotlight systematic, albeit low-grade, immune alterations set in motion by the onset of schizophrenia. Importantly, these immune changes can, over time, exacerbate the risk of schizophrenia by disrupting the structural integrity of both cortical and subcortical regions.

## Supporting information

Liu et al. supplementary material Liu et al. supplementary material

## Supplementary material

The supplementary material for this article can be found at https://doi.org/10.1017/S003329172400014X
